# Low Acrylamide Flatbreads from Colored Corn and Other Flours

**DOI:** 10.3390/foods10102495

**Published:** 2021-10-18

**Authors:** Xueqi Li, Talwinder Kahlon, Selina C. Wang, Mendel Friedman

**Affiliations:** 1 Olive Center, University of California, Davis, CA 95616, USA; spsli@ucdavis.edu; 2 Healthy Processed Foods Research, Western Regional Research Center, Agricultural Research Service, United States Department of Agriculture, Albany, CA 94710, USA; talwinder.kahlon@usda.gov; 3 Department of Food Science and Technology, University of California, Davis, CA 95616, USA

**Keywords:** colored corn flatbreads, quinoa, wheat, peanut meal, beets, broccoli, acrylamide, asparagine, proximate analysis, human health

## Abstract

Dietary acrylamide formed during baking and frying of plant-based foods such as bread and other cereal products, coffee, fried potatoes, and olives is reported to induce genotoxic, carcinogenic, neurotoxic, and antifertility properties in vivo, suggesting the need to keep the acrylamide content low with respect to widely consumed heat-processed food including flatbreads. Due to the fact that pigmented corn flours contain biologically active and health-promoting phenolic and anthocyanin compounds, the objective of this study was to potentially define beneficial properties of flatbread by evaluating the acrylamide content determined by high-performance liquid chromatography/mass spectrometry (HPLC/MS) with a detection limit of 1.8 µg/kg and proximate composition by standard methods of six experimental flatbreads made from two white, two blue, one red, and one yellow corn flours obtained by milling commercial seeds. Acrylamide content was also determined in experimental flatbreads made from combinations in quinoa flour, wheat flour, and peanut meal with added broccoli or beet vegetables and of commercial flatbreads including tortillas and wraps. Proximate analysis of flatbreads showed significant differences in protein and fat but not in carbohydrate, mineral, and water content. The acrylamide content of 16 evaluated flatbreads ranged from 0 to 49.1 µg/kg, suggesting that these flatbreads have the potential to serve as low-acrylamide functional foods. The dietary significance of the results is discussed.

## 1. Introduction

According to McGee [1], flatbreads originated in the Stone Age in parts of the world where grains were the chief food in the ancient diet. Various versions of flatbreads are currently widely consumed in different parts of the world including Greek pita, Indian chapati and roti, Middle Eastern lavash and matzos, Latin American tortillas, and North American johnnycakes. Flatbreads are prepared by baking various doughs without and with added ingredients for about 2 min at temperatures up to ~195 °C. Due to the fact that the exposure of doughs to heat during baking induces the formation of the reactive compound acrylamide reported to cause adverse effects in cells, rodents, and possibly also in humans that include neurotoxicity, reproductive toxicity, genotoxicity, and carcinogenicity [2,3,4,5,6], ongoing worldwide efforts are designed to produce plant-derived baked and fried foods with low acrylamide content. As part of this effort, we previously determined acrylamide content by using a validated chromatography/mass spectrometry method with a detection limit of 1.8 parts-per-billion (µg/kg) of thermally treated olives [7] on 12 new flatbreads using flours prepared from pigmented rice seeds (manuscript in preparation). Due to their low acrylamide levels, these flatbreads might be safe to consume. Moreover, because the blue, red, and yellow colored corn cultivars used to prepare the flatbreads are reported to contain multiple biologically active anthocyanin and phenolic health-promoting compounds [8,9,10,11,12,13,14], the resulting flatbreads have the potential to ameliorate adverse effects of several diseases. We also determined, using standard methods, the proximate composition protein, fat, carbohydrate, dry matter, ash (mineral), and moisture content of the flatbreads. In two related studies, we also previously reported on the development of potentially health-promoting flatbreads with low acrylamide content made by using flours from ancient grains such as brown rice, buckwheat, cornmeal, millet, oats, and quinoa as well as on the acrylamide levels of commercial flatbreads that had been prepared from potato and wheat flours [15,16]. The results show that consumers will be able to select low-acrylamide flatbreads that might also serve as functional foods with high protein and low-fat contents. The main objective of the present study is the further development of novel low-acrylamide flatbreads using white and colored corn flours and flours prepared from mixtures of wheat–quinoa–peanut meal supplemented with beets and broccoli vegetables and to compare the results to acrylamide levels we found in selected commercial tortillas and edible wraps.

## 2. Materials and Methods

### 2.1. Samples

The following corn seeds were purchased online from Purcell Mountain Farms, Moyie Springs, ID, USA (https://purcellmountainfarms.com, accessed on 17 October 2021): Blue Corn, Blue Purcell Corn, Red Corn, White Corn, White Giant Corn, and Yellow Corn. The corn seeds were milled in our laboratory into flours using the Blendtec Kitchen Mill Model 91 at medium setting (Blendtec Inc., Orem, UT 84058, USA). Kirkland Signature quinoa seeds were obtained from the Costco retail store, Richmond, CA, USA, and quinoa flour was prepared in the same manner as the corn seeds. Whole wheat flour was obtained from Bob’s Red Mill (Milwaukie, OR, USA). Peanuts, broccoli, and beets were purchased from local food markets. Peanut oilcake was produced by extracting the oil using Vevor Oil Press (Joyfay.com, Cleveland, OH, USA). The peanut oilcake was ground, and broccoli and beets were chopped by using a Mini-Prep Processor (Cuisinart.com, East Windsor, NJ, USA).

### 2.2. Reagents

Optima LC/MS grade solvents of methanol, acetonitrile, formic acid, hexane and water, and Carrez reagents I and II were all purchased from Fisher Scientific (Waltham, MA, USA). Basix nylon filters (0.45 µm pore size) and disposable syringes were also purchased from Fisher Scientific. Analytical standards of acrylamide (≥99% purity) and acrylamide-d3 solution (500 mg/L in acetonitrile) were obtained from MilliporeSigma (Burlington, MA, USA).

### 2.3. Preparation of Flatbreads

Flatbread dough was prepared by adding 46–49 mL water to 51–54 g of ingredients, as shown in Table 1. Water was added slowly in small volumes to flatbread ingredients until the dough began forming a ball. The dough was kneaded until it became smooth and elastic, which determined the exact amount of water needed for each batch of flatbreads. The dough was placed in a Pyrex bowl, covered with a polyvinyl film, and held at room temperature for 30 min. Dough (50 g) was placed on parchment paper (nonstick, oven-safe up to 216 °C) and pressed into a thickness of 1–1.5 mm and to about 17 cm diameter circle in a 20 cm Alpine Cuisine flatbread Press (Aramco Imports, Inc., Commerce, CA, USA). Flatbreads were cooked between upper and lower hot irons of the flatbread maker for 2 min (1 min each side) at 165–195 °C on parchment paper in a 1000 watt CucinaPro Flatbread Maker (SCS Direct, Inc., Trumbull, CT, USA). The cooking temperature was measured by Fluke 61 Infrared Thermometer (www.fluke.com, accessed on 17 October 2021).

For crispier or chewier flatbreads, cooking time can be adjusted as desired. The pliable flatbreads could be filled and rolled to make wraps. The resulting flatbreads weighed ~32 g before drying and ~20 g after drying.

The compositions of quinoa flour, wheat flour, peanut oilcake, broccoli and beets, and the dough composition of the flatbreads, tested on an as-is basis and prepared from quinoa flour, wheat flour, and peanut oilcake (36%) with broccoli and beets (27%) and salt (0.74%) are shown in our previous publication [17].

### 2.4. Flatbread Proximate Composition Analysis

For proximate as well as acrylamide analysis, cooked flatbreads were chopped for 30 sec in a Cuisinart coffee grinder Model DCG-20N (Cuisinart East Windsor, NJ, USA). Chopped flatbreads were then dried at 103 °C for 3 h. Complete dryness was confirmed with an additional 1 h of drying. Dried flatbreads were ground to fine powders using a coffee grinder (Cuisinart Model DCG-20N E Windsor NJ, USA). Ground flatbreads were analyzed for Kjeldahl nitrogen using AOAC method 990.03 by the Leco FP628 analyzer (Leco Corporation, St Joseph, MI, USA); for crude fat by Soxhlet extraction with petroleum ether using AOAC method 27.006 [18]; for ash using AOAC method 923.03; and for moisture using AOAC method 935.29 [19].

### 2.5. Acrylamide Content of Flatbreads Analysis

Acrylamide in flatbread samples was extracted and analyzed as previously described with minor modifications [15]. To start, 0.5 mL of acrylamide-d3 internal standard (400 µg/L) was added to 2.00 ± 0.01 g of flatbread powder and extracted with 19.5 mL methanol: water (80:20, *v/v*) using a stir plate (Southern Labware, Inc., Cumming, GA, USA) at 300 rpm for 20 min under ambient conditions. Subsequent centrifugation (8000 rpm, 5 min) was conducted on a Sorvall Legend X1 benchtop centrifuge (Thermo Scientific, Waltham, MA, USA) in order to collect supernatant. The supernatant was mixed with 10 mL hexane and centrifuged (4000 rpm, 5 min) again to remove nonpolar fractions. An aliquot (0.1 mL) of Carrez reagents I and II were then added to further eliminate extract turbidity and emulsions. The cleaned supernatant was centrifuged (8000 rpm, 5 min) one more time, and 10 mL of the extract was evaporated to dryness at 35 °C on a Buchi Rotovap R-300 (New Castle, DE, USA). The sample was reconstituted in 1 mL Optima LC/MS grade water and filtered through nylon filters (0.45 µm) prior to HPLC injection.

An isocratic chromatographic separation was conducted on an Agilent C18 Eclipse Plus column (5 µm, 4.6 mm × 250 mm, Santa Clara, CA, USA) using a Waters Alliance 2695 separation module (Milford, MA, USA). Mobile phase A was 0.1% formic acid in water and mobile phase B was formic acid (0.1%) in methanol: acetonitrile (50:50, *v/v*). The column was kept at 25 °C during analysis. The injection volume was 10 µL and the flow rate was 0.4 mL/min for a total of 12 min. The retention times of acrylamide and acrylamide-d3 were 9.01 and 8.92 min, respectively. Sample detection was performed on a Waters Quattro Micro API Mass Spectrometer using multiple reaction monitoring (MRM) in the positive electrospray ionization mode. The source temperature was 120 °C and the desolvation temperature was 400 °C. The capillary voltage was set at 2.75 kV and the cone voltage was 20 V. Desolvation gas and cone gas flow rates were 600 L/h and 25 L/h, respectively. Transitions for acrylamide and acrylamide-d3 were monitored at *m/z* (mass-to-charge ratio) 72 → *m/z* 54.8 and *m/z* 75 → *m/z* 57.9, respectively, with dwell times of 500 msec. A six-point calibration curve of concentration versus the peak area ratio of acrylamide to acrylamide-d3 was used for quantification (R^2^ = 0.9998).

### 2.6. Analysis of Free Asparagine in Corn Flours and Flatbreads by Ion-Exchange Chromatography

The finely ground sample (1.00 g) was measured and placed into a 15 mL glass tube to which sulfosalicylic acid (3%, 10 mL) was added. The tube was covered with parafilm, and the sample was evenly dispersed in the solution by vigorously shaking and kept overnight in refrigerator at 4 °C. The sample was further homogenized the following morning by vigorous shaking. The slurry (1 mL) was then poured into a 1.5 mL Eppendorf centrifuge tube and centrifuged for 15 min. The supernatant solution was then loaded onto an HPLC amino acid analyzer (Biochrom 30+) with an ion exchange column for amino acid separation and post column ninhydrin color derivatization for quantitation. Duplicate analyses were performed using two separate extractions of the same samples, and the method described by Rombouts et al. [20] was used for the determination of acrylamide.

### 2.7. Statistical Analysis

Proximate compositions, as well as flatbread acrylamide and asparagine of flours and flatbreads, were analyzed with Minitab software (version 14.12.0, Minitab Inc., State College, PA, USA) using basic statistics for mean ± standard deviation (SD) and one-way analysis of variance using Tukey’s test. Multiple range test was conducted to determine significant differences among means and (*p* ≤ 0.05) was considered significant.

## 3. Results and Discussion

### 3.1. Proximate Composition of Flatbreads

Table 2 shows the proximate compositions of five flatbreads prepared from two white flours and one each from yellow, red, and blue corn flours. The protein content (in %) ranged from 4.53 (White Giant Corn) to 7 (Red Corn) or a 1.54-fold variation from the lowest to highest value. The corresponding range for crude fat content was from 13.7 (White Corn) to 17.8 (Blue Corn) or a 1.30-fold variation; for ash (mineral) content, the range was from 1.873 (Red Corn) to 2.55 (White Giant Corn) or a 1.36-fold variation; for carbohydrate content, the variation ranged from 36.1 (Blue Corn) to 43.5 (Red Corn) or a 1.20-fold variation; and for water content, the range was from 33 (Red Corn) to 39.4 (White Giant Corn) or a 1.19-fold variation. The results indicate that although the data show significant differences among each of the six evaluated proximate components in the corn-based flatbreads, the Red Corn flatbreads have the highest protein content, the Blue Corn flatbreads have the highest crude fat content, and the White Giant Corn flatbreads have the highest mineral content. These data offer a choice to consumers who might wish to select flatbreads that best meets their dietary needs for protein, fat, carbohydrates, and mineral content and facilitate labeling the content by manufacturers. 

Table 3 shows the proximate composition on a dry matter (DM) basis of corn flours used to prepare the flatbreads. The protein content ranged (in %) from 5.19 (White Giant Corn) to 8.65 (Red Corn) or a 1.67-fold variation from the lowest to highest value. The corresponding range of crude fat was from 10.4 (White Corn) to 14.0 (Blue Corn) or a 1.35-fold variation; for mineral content, the range was from 1.18 (Blue Corn) to 1.51 (White Giant Corn) or a 1.28-fold variation; for total carbohydrate, the range was from 67.8 (Red Corn) to 70.06 (White Giant Corn) or a 1.03-fold variation; for dry matter, the range was from 88.39 (Yellow Corn) to 90.15 (White Corn) or a 1.02-fold variation; and for water, the range was from 9.85 (White Corn) to 11.61 (Yellow Corn) or a 1.18-fold variation. These results show that the protein and fat contents seem to vary significantly among the six corn flours and that the corresponding variations in carbohydrate, mineral, and water content is minor.

We previously reported [17] the composition of four flatbreads prepared from the following flour mixtures (combinations): quinoa–peanut meal–broccoli; wheat–peanut meal–broccoli; quinoa–peanut meal–beet; and wheat–peanut meal–beet (Table 4). The protein composition range was quite narrow ranging (in % dry matter basis) from 29.55 to 32.8. The corresponding range for fat content was from 4.71 to 5.80 or 1.23-fold variation from lowest to highest value. The range for ash (mineral) content was from 4.14 to 4.40 or a 1.06-fold variation. The carbohydrate content ranged from 21.69 to 27.51 or a 1.06-fold variation. The concentration ranges for dry matter and water were also quite narrow. These composition data are mentioned here because we determined the acrylamide content of these flatbreads in the present study.

### 3.2. Acrylamide Content of Flatbreads

#### 3.2.1. Accuracy, Precision, and Sensitivity of the Analytical Method for Acrylamide

**Figure 1:**
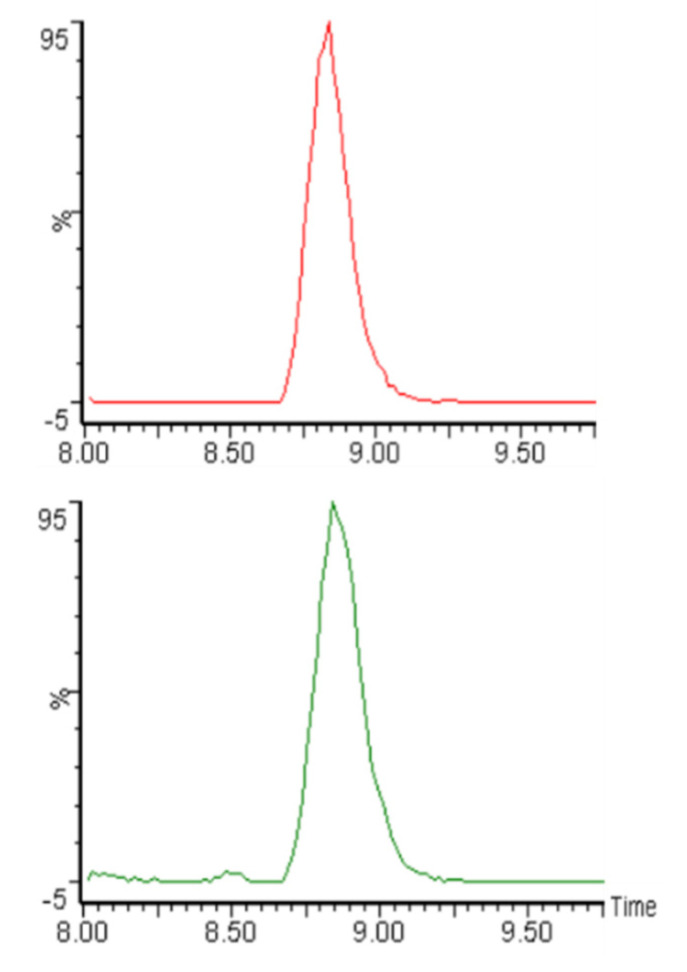
An HPLC-MS/MS chromatogram example of acrylamide-d3 (**top**) and acrylamide (**bottom**) in the Red Corn flatbread. Y-axis: intensity; X-axis: retention time (min).

Each type of flatbread was prepared in triplicate, and acrylamide of each replicate was determined. Figure 1 shows an example of HPLC-MS/MS chromatograms of the internal standard (acrylamide-d3) and of acrylamide in the Red Corn flatbread that demonstrate the sensitivity of the assay. Similar chromatograms were obtained for acrylamide in all the other flatbreads. One white corn flatbread replicate was randomly selected for routine analytical method validation. The recovery of acrylamide was calculated by spiking this white corn flatbread replicate with known amount of acrylamide standard solution (50 µg/kg). A recovery rate of 90% was achieved. Intra-day and inter-day precision analyses were carried out using the same white corn flatbread replicate. Intra-day relative standard deviation (RSD) was calculated from running this replicate three times on the same day, while inter-day RSD was calculated from running this replicate once per day over three consecutive days, which yielded values of 1.2% and 4.5%, respectively. The limit of detection and the limit of quantification of the analytical method were determined as 3 and 10 times the signal-to-noise ratio at 2.1 µg/kg and 7.1 µg/kg, respectively. The detection limit is estimated at 1.8 µg/kg, similar to that we reported in two previous studies [15,16]. The protein, fat, carbohydrate, and ash content of the samples did not seem to affect the extractions analysis for acrylamide.

#### 3.2.2. Corn-Based Flatbreads

**Figure 2:**
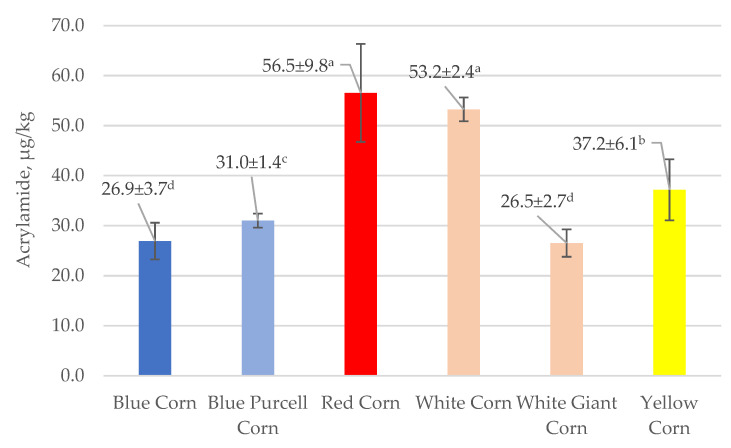
Acrylamide in colored corn flatbreads, µg/kg. Values are mean ± SD. Values with different superscript letters differ significantly (*p* ≤ 0.05). Y-axis, acrylamide µg/kg; X-axis, experimental and commercial* flatbreads.

Figure 2 shows that the range of acrylamide levels of flatbreads prepared from two white and four colored flours ranged from 26.5 (White Giant Corn) to 56.5 (Red corn) or a 2.1-fold variation from lowest to highest value. What is noteworthy is the two-fold variation in the acrylamide content of White Giant Corn (26.5) and White Corn (53.2), suggesting that acrylamide levels of flatbreads from different varieties of white corn seeds might not be similar. Acrylamide levels in the flatbreads correlated (Pearson Correlation coefficient) positively with the carbohydrate (0.967, *p* < 0.05) and negatively with the water (−0.935, *p* < 0.05) content in the finished product. Comparisons of the starting material to the finished product showed that acrylamide content in the finished flatbreads was negatively correlated (−0.821, *p* < 0.05) with the fat content of the flours. We do not know the chemical basis for the observed correlation. The high positive value of the correlation of acrylamide and carbohydrate values is likely due to a commensurate level of reducing sugars such as glucose, a precursor of acrylamide. [21].

The data in Table 3 and Figure 2 show that the flatbreads prepared using Red Corn flour with the highest protein content have the highest acrylamide concentration and that the other corn flatbreads with lower protein content have lower levels. In addition, flatbreads prepared from Blue Corn flour with the highest fat content have the lowest acrylamide concentration. These results contribute to our insight about the possible relationships of composition to acrylamide content. 

Overall, the data suggest that the highest level of acrylamide shown in Figure 2 is four to eight times lower than compared to the reported acrylamide content of a large number of commercial baked products sold in Italy [22]. It seems, therefore, that these low-acrylamide, gluten-free flatbreads might be safe to consume. Moreover, the phenolics and flavonoids present in pigmented rice flours and the prepared flatbreads, the subject of a future study, are expected to contribute additional health benefits to the low-acrylamide, gluten-free flatbreads.

#### 3.2.3. Acrylamide Content of Flatbreads from Wheat–Quinoa–Peanut Meal Flours with Added Beets or Broccoli and of Commercial Tortillas Wraps

**Figure 3:**
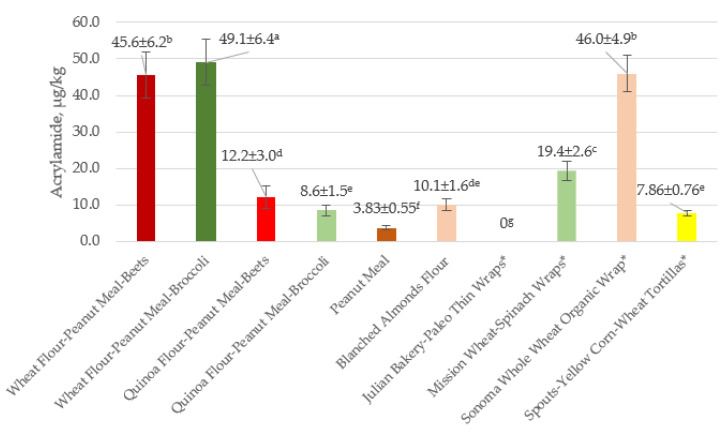
Acrylamide in quinoa and wheat experimental and commercial* flatbreads, µg/kg. Values are mean ± SD. Values with different superscript letters differ significantly (*p* ≤ 0.05). Y-axis, acrylamide µg/kg; X-axis, experimental and commercial* flatbreads.

In order to place the results of the present study in perspective, it is instructive to compare the acrylamide content of several commercial flatbreads, including tortillas, to the values of experimental flatbreads prepared in the present study. Figure 3 shows that the acrylamide levels of four commercial products marked with an asterisk ranged from 0 (Julian Baker-Paleo Thin Wraps) to 46.0 (Sonoma Whole Wheat Organic Wrap). The acrylamide contents of four experimental peanut meal flatbreads were not correlated with proximate analysis. The values for the six experimental flatbreads ranged from 3.83 (Peanut Meal) to 49.1 (Wheat Flour–Peanut Meal Broccoli). It seems that the added wheat induced an increase in the acrylamide concentration of the peanut meal by an unknown mechanism. We previously observed similar increases in acrylamide content of flatbreads by added fruit and vegetable peels [16].

Overall, these results suggest that both commercial wraps and tortillas and the experimental flatbreads contain low levels of acrylamide ranging from ~3.8 to 56.5 ug/kg (Figure 2 and Figure 3). However, unlike the experimental corn flatbreads, wheat-based commercial wraps and tortillas and the experimental flatbreads all contain gluten proteins, which might prevent them from being consumed by some individuals who are allergic to or suffer wheat-gluten-induced adverse effects.

#### 3.2.4. Reported Acrylamide Content of Tortillas

Here, we briefly mention the contrasting results in two published studies on some of the factors that affect acrylamide levels of tortillas. Due to the fact that acrylamide can be formed during the process of frying tortilla chips in oils, Topete-Betancourt et al. [23] determined the acrylamide levels formed during three different nixtamalization processes—traditional, ecological, and classic—used with tortillas. They found large variations in the acrylamide levels depending on the frying process ranging from 46.3 to 1443.4 µg/kg or a 31.2-fold variation from lowest to highest value. The authors suggest that differences in acrylamide values seem to be related to the effect of different cations (Ca^2+^, Mg^2+^, Fe^2+^, Zn^2+^, Na^+^, and K^+^) present in wood ashes, lime, and salt used as raw material and that nixtamalization seems to be an effective and inexpensive method for acrylamide mitigation. The lowest value seems similar to the value we found in the commercial tortillas. It is also of interest that the detection limit of 20 µg/kg observed with the HPLC-UV method used to determine acrylamide levels is about 11 times higher than the corresponding limit of 1.8 µg/kg in the present study that used an HPLC/MS method [15,16]. In a related study, Uscanga-Ramos et al. [24] evaluated the effect of pre-fry drying on the formation of acrylamide determined by GC-MS in corn tortillas chips fried in ten batches. The results show that all acrylamide concentrations were below the detection limit of 20 µg/kg, suggesting that the type of oil used in frying, including avocado oil, did not seem to affect acrylamide content. Producers of tortillas should select a process that produces tortillas with low acrylamide content.

These cited studies imply that the described flatbreads prepared from milled colored seeds and that are rich in phenolic and flavonoid compounds have the potential to serve as functional foods that might ameliorate the adverse effects of multiple diseases.

#### 3.2.5. Asparagine Content of Corn Flours and Flatbreads

Table 5 shows that the concentration of the asparagine content of the six corn flours (in µg/g) ranged from 466 (Red Corn) to 1544 (White Giant Corn) or a 3.3-variation from the lowest to the highest value. The corresponding range for the flatbreads made with these flours was from 391.5 (Red Corn) to 1331 (White Giant Corn) or a similar 3.4-fold variation. The data also show that both white corn flour and corresponding flatbreads had much higher asparagine content than both the colored flours and corresponding flatbreads. What is also of interest is the apparent lower amounts of asparagine in flatbreads than in flours, presumably because the asparagine in flours had been destroyed during thermal processing, as described in detail elsewhere [2,25]. Note the wide variation in the decrease in the six corn flatbreads. Moreover, the presence of asparagine in the flatbreads implies that higher temperatures and longer baking times than those used in the present study would likely result in increased acrylamide content.

The analysis of asparagine and other free amino acids was determined by HPLC with the aid of the ninhydrin chromophore for which its mechanism is described elsewhere [26]. Only asparagine results are included in the present paper. In previous related studies, we used the HPLC method to determine free amino acids in fruits and vegetables following derivatization with a different chromophore [27,28,29,30]. Since we found that the free amino acid content changed during different stages of growth of the plants, it would be of interest to determine if this is also the case during the growth of corn kernels and other cereals. Corn harvested during a growth stage with low levels of asparagine would have the potential to produce low amounts of acrylamide during baking to make corn flatbreads, corn breads, and corn tortillas.

The following additional observations on asparagine/acrylamide relationships reflect different experimental approaches designed to reduce the acrylamide content of the diet. The calculated negative correlation coefficient (−0.536) of acrylamide content of flatbreads (Figure 2) versus asparagine content of flours (Table 5) implies that acrylamide content increases with decreasing asparagine content. Žilić et al. [21] also found that the asparagine content of flours, including blue corn, affected the acrylamide content of biscuits. A related study [31] found that different commercial brands of corn products had wide-ranging amounts of acrylamide (<30–410 µg/kg), suggesting that consumers have a choice of selecting commercial corn-based and other foods with low acrylamide content. These include flatbreads prepared from corn flour as well as almond flour, peanut meal, wheat flour, quinoa flour–peanut meal, wheat flour–spinach, wheat flour–peanut meal–beets, wheat flour–peanut meal–broccoli, yellow corn–wheat tortillas, and wraps ((26.5–56.5 µg/kg, Figure 2) and (3.83–49.1 µg/kg, Figure 3)).

Since the levels of asparagine as a proportion of the total free amino acid pool seem to be a key component in acrylamide formation, Muttucumaru et al. [32] suggest that plant genetic studies might reduce the asparagine content of plant foods by blocking enzymes involved in its biosynthesis. A complementary approach involves postharvest treatment of plant foods before thermal processing with the enzyme asparaginase, which catalyzes the hydrolysis of asparagine to aspartic acid and ammonia [31]. These promising enzymatic methods merit study with corn and other cereal seeds.

## 4. Conclusions

Food processing is used to make food edible, to alter flavor and texture, and to destroy pathogenic microorganisms and toxins. The processing methods include baking, broiling, cooking, and roasting. The exposure of foods to these processing methods have both beneficial and harmful effects that include the formation of potentially toxic acrylamide. This study and cited earlier studies show that flatbreads prepared from ancient grains that include millet, oat, quinoa, rye, and sorghum and from pigmented (colored) corn flours seem to have low acrylamide levels, suggesting that they might be safer to consume than processed foods with much higher level and might, therefore, be considered as health-promoting functional foods. In addition, because the flatbreads prepared from colored grains contain health promoting phenolic and anthocyanin compounds, they also might ameliorate adverse effects associated with various diseases. Flatbreads prepared from grains other than wheat with a high-protein and low-fat content as determined in the present study by proximate analysis also provide consumers with a gluten-free and nutritious readily available food choice. Finally, because the amino acids tryptophan and lysine are nutritionally limiting in corn [33], the preparation and nutritional evaluations of flatbreads, including widely consumed tortillas from high-tryptophan and high-lysine colored corns [33], also merit study.

In conclusion, flatbreads prepared from colored corn and other flours have low acrylamide content and contain antioxidative phenolic and anthocyanin compounds. Therefore, they represent new functional foods that merit commercial production and sale by food processors, as well as preparation and consumption in restaurants and in homes.

## Figures and Tables

**Table 1:** Flatbread dough composition.

Flatbread	Flour, g	Guar Gum, g	Salt, g	Water, mL	Olive Oil, g
Blue Corn	200	8	2.2	200	8
Blue Purcell Corn	200	8	2.2	190	8
Red Corn	200	8	2.2	190	8
White Corn	200	8	2.2	200	8
White Giant Corn	200	8	2.2	210	8
Yellow Corn	200	8	2.2	200	8
Blanched Almond Flour	150	9	1.59	100	0
Peanut Meal	150	9	1.59	130	0

**Table 2:** Proximate composition of flatbreads made from colored corn on a dry matter basis. Shown values are in % ^1^. Values with different superscript letters differ significantly (*p* ≤ 0.05).

Flatbread	Protein (N × 6.25), g	Crude Fat, g	Ash, g	Total Carbohydrate, g	Moisture Content of Flatbreads, as Is	
					Dry Matter, g	Water, mL
Blue Corn	5.6 ± 0.2 ^cd^	17.8 ± 0.8 ^a^	2.07 ± 0.02 ^b^	36.1 ± 0.3 ^e^	61.6 ± 0.6 ^cd^	38.4 ± 0.6 ^bc^
Blue Purcell Corn	6 ± 1 ^b^	16.2 ± 0.3 ^b^	2.06 ± 0.02 ^b^	36.4 ± 0.5 ^e^	60.9 ± 0.6 ^de^	39.1 ± 0.6 ^ab^
Red Corn	7 ± 1 ^a^	14.9 ± 0.7 ^c^	1.873 ± 0.008 ^e^	43.5 ± 0.6 ^a^	67 ± 2 ^a^	33 ± 2 ^e^
White Corn	5.9 ± 0.2 ^bc^	13.7 ± 0.8 ^d^	1.96 ± 0.03 ^a^	42.9 ± 0.3 ^b^	64 ± 1 ^b^	36 ± 1 ^d^
White Giant Corn	4.53 ± 0.05 ^d^	15.9 ± 0.3 ^b^	2.55 ± 0.02 ^c^	37.6 ± 0.1 ^d^	60.6 ± 0.5 ^e^	39.4 ± 0.5 ^a^
Yellow Corn	5.071 ± 0.008 ^d^	15 ± 2 ^c^	2.04 ± 0.02 ^c^	39.8 ± 0.6 ^c^	62 ± 1 ^cd^	38 ± 1 ^b^

**^1^** Analysis was conducted in triplicate. Values are mean ± SD. Total Carbohydrate = (dry matter–Protein–crude fat–ash). Values with different superscripts differ significantly (*p* ≤ 0.05).

**Table 3:** Proximate composition of colored corn flours on a dry matter basis. Shown values are in % ^1^. Values with different superscript letters differ significantly (*p* ≤ 0.05).

Corn Flour	Protein (N × 6.25), G	Crude Fat, g	Ash, g	Total Carbohydrates, G	Dry Matter Basis, g	Water, mL
Blue Corn	6.43 ± 0.02 ^d^	14.0 ± 0.1 ^a^	1.18 ± 0.01 ^d^	68.3 ± 0.2 ^d^	89.91 ± 0.09 ^b^	10.1 ± 0.3 ^d^
Blue Purcell Corn	6.49 ± 0.01 ^c^	12.4 ± 0.2 ^b^	1.31 ± 0.01 ^b^	69.6 ± 0.3 ^c^	89.80 ± 0.07 ^c^	10.20 ± 0.07 ^d^
Red Corn	8.65 ± 0.06 ^a^	11.0 ± 0.4 ^e^	1.26 ± 0.04 ^c^	67.8 ± 0.4 ^e^	88.75 ± 0.05 ^e^	11.25 ± 0.05 ^b^
White Corn	6.82 ± 0.06 ^b^	10.4 ± 0.2 ^f^	1.19 ± 0.03 ^d^	71.8 ± 0.2 ^a^	90.15 ± 0.02 ^a^	9.85 ± 0.02 ^e^
White Giant Corn	5.19 ± 0.05 ^f^	12.2 ± 0.3 ^c^	1.51 ± 0.04 ^a^	70.06 ± 0.07 ^b^	88.9 ± 0.1 ^d^	11.1 ± 0.1 ^c^
Yellow Corn	5.81 ± 0.04 ^e^	11.5 ± 0.3 ^d^	1.16 ± 0.01 ^d^	70.0 ± 0.3 ^b^	88.39 ± 0.02 ^f^	11.61 ± 0.02 ^a^

^1^ Analysis was conducted in triplicate. Values are mean ± SD. Total Carbohydrate = (dry matter–protein–crude fat–ash).

**Table 4:** Composition of quinoa, wheat, and peanut meal with broccoli or beet flatbreads, % DM basis. Composition of quinoa, wheat, and peanut meal with broccoli or beet flatbreads, % dry matter basis. Values are mean ± SD, *n* = 3. Adapted from Kahlon et al. [17]. Values with different superscript letters differ significantly (*p* ≤ 0.05).

Flatbreads	Protein (N × 6.25), g	Fat, g	Ash, g	Total Carbohydrates, g	Dry Matter Basis, g	Water, mL
Quinoa–peanut meal–broccoli	32.81 ± 0.01 ^a^	5.80 ± 0.06 ^a^	4.40 ± 0.01 ^a^	21.69 ± 0.03 ^d^	64.7 ± 0.3 ^d^	35.3 ± 0.3 ^a^
Wheat–peanut meal–broccoli	30.59 ± 0.06 ^c^	4.71 ± 0.03 ^c^	4.25 ± 0.02 ^c^	26.01 ± 0.04 ^c^	65.6 ± 0.5 ^c^	34.4 ± 0.5 ^b^
Quinoa–peanut meal–beets	31.95 ± 0.05 ^b^	5.3 ± 0.4 ^b^	4.35 ± 0.01 ^b^	27.1 ± 0.2 ^b^	68.7 ± 0.3 ^a^	31.3 ± 0.3 ^d^
Wheat–peanut meal–beets	29.55 ± 0.02 ^d^	5.15 ± 0.07 ^b^	4.14 ± 0.01 ^d^	27.51 ± 0.03 ^a^	66.35 ± 0.08 ^b^	33.65 ± 0.08 ^c^

**Table 5:** Average asparagine values with means and standard deviations from two separate extractions of colored corn flours and flatbreads.

Sample	Asparagine µg/g ^1^
Flours	
Blue Corn	675.0 ± 4.2
Blue Purcell Corn	756.5 ± 6.4
Red Corn	466 ± 25
White Corn	756.0 ± 8.5
White Giant Corn	1544 ± 64
Yellow Corn	624 ± 16
Flatbreads	
Blue Corn	651.5 ± 7.8
Blue Purcell Corn	686 ± 16
Red Corn	391.5 ± 9.2
White Corn	559 ± 14
White Giant Corn	1331 ± 59
Yellow Corn	463 ± 25

^1^ Analysis were in duplicate, *n* = 2.

## Data Availability

Data are available upon request.
